# Intersession Intra-Rater and Inter-Rater Reliability of Myotonometer for Upper and Lower Extremity Muscles in Children with Spinal Muscular Atrophy

**DOI:** 10.3390/diagnostics14202300

**Published:** 2024-10-16

**Authors:** Seval Kutlutürk Yıkılmaz, Tülay Çevik Saldıran, Özgül Öztürk, Sedat Öktem

**Affiliations:** 1 Department of Physiotherapy and Rehabilitation, Faculty of Hamidiye Health Sciences, University of Health Sciences, 34668 Istanbul, Turkey; 2 Department of Physiotherapy and Rehabilitation, Faculty of Health Sciences, Bitlis Eren University, 13000 Bitlis, Turkey; tlyfztcvk@gmail.com; 3 Department of Physiotherapy and Rehabilitation, Faculty of Health Sciences, Acıbadem Mehmet Ali Aydınlar University, 34752 Istanbul, Turkey; ozgul.ozturk@acibadem.edu.tr; 4 Department of Pediatric Pulmonary Diseases, Faculty of Medicine, Istanbul, Istanbul Medipol University, 34810 Istanbul, Turkey; soktem@medipol.edu.tr

**Keywords:** muscle tone, myotonometry, muscle stiffness, reliability, spinal muscular atrophy

## Abstract

**Background/Objectives:** This study aimed to examine intra- and inter-rater reliability of a myotonometer (MyotonPRO) in measuring upper and lower extremity mechanical properties in children with spinal muscular atrophy types I and II. **Methods:** Biceps brachii, triceps brachii, rectus femoris, and gastrocnemius muscle tone and stiffness in children (*n* = 21) were measured using the MyotonPRO device. Examiner 1 performed two sets of measurements in 60 min to determine intra-rater reliability. Examiner 2 performed measurements between Examiner 1’s sets. Intra–interclass correlation coefficient, minimal detectable change, and standard error of measurement values were calculated to assess intra- and inter-rater reliabilities in this cross-sectional study. **Results:** The results showed excellent intra- and inter-rater reliability analyses for frequency and stiffness values except for the stiffness value of the gastrocnemius muscle, which presented good reliability (ICC = 0.71). Minimal detectable change values ranged from 0.59 to 1.98 Hz for muscle tone and 16.08 to 124.74 N/m for stiffness (for both intra- and inter-rater reliabilities). **Conclusions:** Our findings indicate that MyotonPRO is a reliable tool for quantifying upper and lower extremity mechanical properties within one session in children with spinal muscular atrophy types I and II. Mechanical properties of the extremity muscle can be determined using this easily applied tool in future studies.

## 1. Introduction

Spinal muscular atrophy (SMA) is a rare hereditary neurodegenerative disease and is also one of the most common neuromuscular diseases caused by the degeneration of spinal cord motor neurons [1]. Low muscle tone is one of the most common clinical features observed in children with SMA. Areflexia, progressive skeletal muscle weakness, bulbar weakness, scoliosis and fatigue are other common features observed in untreated children with SMA [2,3,4,5].

The internal tension of skeletal muscle is influenced by both neuronal and non-neural factors, both of which have the potential to cause abnormal muscle tone [5]. The tonic stretch reflex is produced when a muscle is assisted by neural commands from cortical and subcortical regions, spinal circuitry, a stretch reflex, and/or other peripheral inputs, and is the representation of the neural component of muscle tone [6]. The abnormal muscle tone results in imbalanced movement patterns, making it challenging to control muscle movements. This can lead to reduced muscle strength, improper alignment, and delays in the development of motor skills [7]. Persistent hypotonia causes poor joint stability, poor postural alignment, decreased activity tolerance, and delayed motor skill acquisition [8]. Considering the experienced difficulties with muscle tone in this population, an accurate assessment of muscle mechanical properties can help establish the diagnosis, plan appropriate treatment methods, and follow the prognosis of the disease.

Identifying skeletal muscle mechanical properties with a myotonometer as an objective evaluation tool has increased over time. Myotonometers can quantify muscle tone and the level of severity of hyper/hypotonic conditions [9,10,11,12]. The modified Tardieu scale (MTS) and modified Ashworth scale (MAS) are commonly utilized in clinical settings to evaluate muscle tone [13,14,15]. The widely used MAS has been criticized for its inability to differentiate between increased muscle tone and soft tissue stiffness [16,17,18]. Myotonometers can serve as a reliable tool for measuring the mechanical properties (muscle tone and stiffness) of different skeletal muscles [19]. Skeletal muscle resting tone can be defined as the resting internal tension of the muscle tissue or the resistance to passive stretch that reflects the relative influences of muscle-elastic characteristics and neural drive to the muscle [10]. Stiffness refers to the resistance of soft tissue to an external force and is calculated using the damped natural oscillation response [2,12,20]. Previous studies have demonstrated the reliability, validity, and accuracy of the myotonometer [17,18,21,22,23]. Myotonometers were used to investigate the effectiveness of treatment on mechanical properties of skeletal muscle in pediatric population studies, especially in newborns, [10] Duchenne Muscular Dystrophy, [24] and cerebral palsy [9,25]. The effectiveness of Vojta therapy applied for four weeks on the back extensor muscles of newborns was objectively demonstrated with a myotonometer [10]. However, only a cross-sectional study was conducted in children with SMA Type III [11].

Myotonometer examination has not yet been conducted on children with SMA Types I and II to determine the mechanical characteristics of the muscles in the upper and lower extremities. Therefore, examining muscle mechanical properties in individuals with SMA may have important implications for potential peripheral mechanisms. However, to observe these activities with a myotonometer, the reliability of use in individuals with SMA should be questioned in the first stage. Accordingly, this study aimed to examine the intra- and inter-rater reliabilities of MyotonPRO in measuring upper and lower extremity muscle tone and mechanical properties in individuals with SMA Types I and II.

## 2. Materials and Methods

This study was approved by Istanbul Medipol University, Non-Interventional Clinical Research Ethics Committee with the Number E-10840098-772.02-3771. Written consent was obtained from the parents of the children, and all procedures were conducted according to the Declaration of Helsinki. The study was registered on ClinicalTrials.gov (*NCT05521217).

### 2.1. Design and Participants

**Figure 1:**
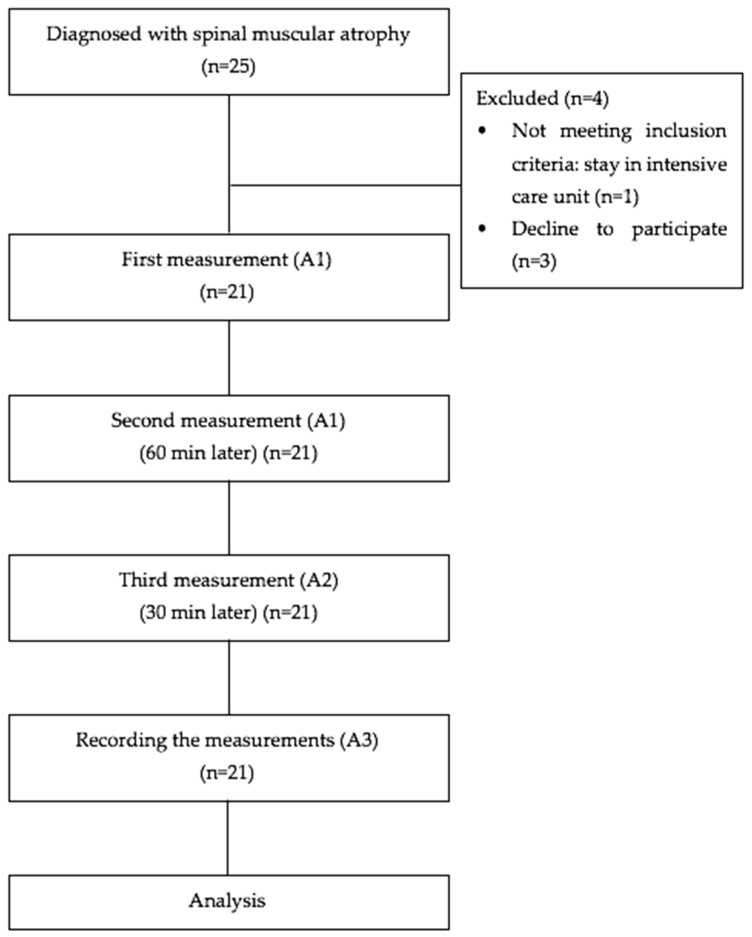
Flowchart of the study.

The children with SMA who were followed at Bağcılar Medipol Mega University, Pediatric Pulmonology Clinic were included in the study between July 2022 and December 2022. The inclusion criteria consisted of several parameters: (i) clinically and genetically diagnosed with Type I or II SMA, (ii) age range of 1 to 4 years, (iii) child can be removed from respiratory support during the measurements and easily tolerates the measurements, (iv) child receiving medical treatments (Nusinersen, Evrysdi, Zolgensma), and (v) child living in a home environment and not in intensive care. Exclusion criteria consisted of several parameters: (i) surgery in the last six months, (ii) implant placement within the extremities, (iii) plagiocephaly, advanced scoliosis, hip dislocation, and/or other neurological and orthopedic disorders that may affect motor functions, and (iv) unwillingness of families to participate in the study (Figure 1). The participants in the study were continuing their regular physiotherapy and rehabilitation program and were not using any medication that could alter their muscle tone.

Information on age, gender, age at diagnosis, type of SMA, treatments, feeding status, presence of tracheostomy, usage of cough assist, and bilevel positive airway pressure (BIPAP) use were recorded on the demographic and clinical evaluation form. 

### 2.2. Evaluation of Motor Functions

The Children’s Hospital of Philadelphia Infant Test of Neuromuscular Disorders (CHOP-INTEND) was used to evaluate motor functions. It is a disease-specific scale for evaluating patients with neuromuscular diseases, including those with SMA. The development of the tool was based on the progression of motor function. The test includes 16 items that provide information on muscle strength and function with and against gravity. Items are ranked from easy to difficult with the least tolerated items undergoing testing. The scoring is graded from 0 (no response) to 4 (complete response). The maximum score is 64. The test can be completed in 15 to 40 min using some types of equipment (mat, rattle, plush toy, toy phone) [26].

### 2.3. Measures

Evaluation of muscle mechanical properties was performed using the MyotonPRO (Myoton AS., Tallinn, Estonia) device. The external force applied from the device probe causes displacement in the examined tissue. Determination of muscle mechanical properties can be made by calculating oscillatory displacements. The oscillation frequency (Hz), and stiffness (N/m) values are measured with the device. The transducer captures the acceleration of the muscle’s oscillatory response and determines the oscillation frequency (Hz). This frequency of oscillations is calculated using the formula Hz = 1/T, where T is the oscillation period in seconds. The oscillation frequency (Hz) represents the resting internal intramuscular tension (muscle tone). A higher score indicates more intramuscular tension [17,18]. Stiffness (N/m) is a biomechanical property of the tissue that can be defined as the resistance to external forces that will cause the tissue to change its shape [19].

During the MyotonPRO application, the examiner grasps the device and applies pressure at a right angle to the muscle. The MyotonPRO probe applies a brief mechanical impulse (0.18 N, 15 ms) to the skin to record different oscillation parameters of the muscle response. This mechanical impulse at a predetermined force (0.4 N) follows a quick release and induces dampened natural oscillations of the muscle [20,27]. With the application of pressure, the inner probe is pushed into the muscle until the plexiglass frame is immobilized on the skin’s surface. From the marked estimated motor point, the pressure application was terminated when the red light on the plexiglass frame of the device probe turned green, and the probe was kept constant until five pulses were completed. The duration of one pulse was set at 15 ms, and the interval between pulses was set at 8 ms. The device records these oscillations in the form of an acceleration graph and calculates parameters using values obtained from the graph. Detailed information can be found in the user guide of the device [28].

### 2.4. Procedure

Before starting the MyotonPRO assessment, demographic information related to children was recorded on the data entry platform of the software program. Pattern entries, which determine that all measurements will be made from the estimated motor point of the relaxed muscles in the resting position, were loaded into the system. Measurements were performed bilaterally from the rectus femoris muscle for knee extensors, biceps brachii muscle for forearm flexors, triceps brachii muscle for forearm extensors, and gastrocnemius muscle for ankle plantar flexors. These muscles were selected because they play an important role in daily living activities and the functional mobility of children with spinal muscular atrophy. In addition, as the number of muscles measured increases, children’s participation in the measurement may be affected, so the measurements were limited to 2 muscles in the upper extremity and 2 muscles in the lower extremity. Measurements were performed 2 h after feeding in a quiet, well-lit room with a neutral temperature. The children were calm, and most of their clothing was removed. The SENIAM sensor location guideline was considered as a reference for the estimated motor point determination in the MyotonPRO measurement [29]. Positions during the assessments are listed below:

- Rectus femoris measurement: The children were positioned sitting in a chair with the knees slightly flexed and the upper body leaning slightly back. The midpoint (50%) of the line extending from the anterior spina iliaca superior to the patella superior was marked as the evaluation point.
- Biceps brachii measurement: The children were supported in a sitting position with the elbow joint in 90° flexion and the forearm in supination. The reference line was determined in the direction of the line between the acromion and the fossa cubiti. The distance of 1/3 of this length from the fossa cubiti reference was accepted as the estimated reference motor point.
- Triceps brachii measurement: The children were supported in a sitting position with the shoulder joint in 90° abduction, elbow 90° flexion, and forearm supinated. The line between the posterior cristae of the acromion and the olecranon was determined as the reference line. The midpoint (50%) of this line was marked as the predicted motor point.
- Gastrocnemius measurement: The children were placed in a prone position. The most prominent protrusion of the muscle was marked as the estimated motor point while the dorsum of the foot was supported at the table corner. The same measurements were made while the children with a tracheostomy were in a side-lying position.

All assessments were performed bilaterally by two researchers (A1 and A2) who have experience in using the myotonometer device. Both researchers performed the measurement procedures in the same order. After standard positioning was achieved, the measurement was taken by placing the device probe at a right angle to the estimated motor points by A1 and repeating the same measurements 60 min after the first session. A2 took measurements from the estimated motor points 30 min after the A1 measurement was obtained. A1 and A2 were blinded to their previous measurement results by exporting the first session results to the computer and removing them from the device. The examiners were not aware of each other’s results. The measurement results of both researchers were recorded by the third researcher (A3) (Figure 1). All assessments were conducted in the same session, and there was a rest period between the measurements to prevent fatigue. All measurements, including rest intervals, were completed within 3 h.

### 2.5. Statistical Analysis

Data analysis was performed using SPSS 22.0 software (IBM Corp., Armonk, NY, USA). The normality of the data was assessed with the Shapiro–Wilk test and skewness–kurtosis values. Demographic and clinical characteristics were shown with descriptive analysis by the mean and standard deviation (SD) or number and percentages (%). Intra-rater (between sessions) and inter-rater reliability were assessed by calculating intraclass correlation coefficient (ICC) values with 95% confidence intervals (CI). ICC values > 0.75 were considered excellent, between 0.74 to 0.40 as good to fair, and <0.40 as poor reliability [30]. Standard error of measurement (SEM), minimal detectable change (MDC), and 95% limits of agreement (LOA) were calculated to assess absolute reliability. For SEM calculations, the SEM = SD*√1 − ICC formula was used which indicates standard deviation. MDC was calculated with the formula: MDC = SEM × √2 × 1.96 [31]. The LOA was calculated by multiplying the SD of the mean difference (MD) with 1.96 and adding it to or subtracting it from the MD. Systematic bias between measurements was identified using Bland–Altman plots. No data were missing.

We determined the required sample size by considering an ICC of 0.70 with two raters at an α level of 0.05 and power of at least 80%, and at least 19 participants were needed for this study [32]. A total of 21 participants were evaluated to ensure an assumption of possible data loss.

## 3. Results

### 3.1. Demographics

Twenty-one participants with SMA type I or type II were recruited in the study. The demographic and clinical characteristics of the participants are presented in Table 1.

### 3.2. Intra-Rater Reliability

For intra-rater reliability analysis, the ICC values for the frequency and stiffness parameters of the studied muscles were above 0.75, which indicates excellent reliability (Table 2 and Table 3). SEM and MDC values are provided in Table 2 and Table 3.

The SEM of all evaluated muscles ranged from 0.38 to 0.71 Hz for muscle tone and 11.28 to 45.00 N/m for stiffness. The MDC of both muscles ranged from 1.07 to 1.98 Hz for muscle tone and 31.29 to 124.74 N/m for stiffness. Upper and lower LOA values and mean differences for intra- and inter-rater analyses are presented in Table 4.

According to the Bland–Altman plots for intra-rater reliability analysis of the frequency values, among the evaluated lower extremity muscles, the right side rectus femoris muscle and left and right side gastrocnemius muscles showed systematic bias (Figure S1). Except for these muscles, the frequency parameters showed no systematic bias for both sides of the biceps brachii, triceps brachii, and left-side rectus femoris muscles (Figure S1). The Bland–Altman plots of stiffness values for the intra-rater analysis showed no systematic bias for right-side biceps brachii and right and left-side triceps brachii muscles (Figure S2). Except for these muscles, other assessments showed systematic bias (Figure S2).

### 3.3. Inter-Rater Reliability

Except for the stiffness value of the left gastrocnemius muscle which presented good reliability (ICC = 0.71), the ICCs for the inter-rater reliability analysis were above 0.75 and showed excellent reliability. A summary of the ICCs with the 95% CI values is presented in Table 2 for frequency and in Table 3 for stiffness parameters. SEM and MDC are provided in Table 2 and Table 3.

The SEM of all evaluated muscles ranged from 0.21 to 0.62 Hz for muscle tone and 5.80 to 36.83 N/m for stiffness. The MDC of both muscles ranged from 0.59 to 1.74 Hz for tone and 16.08 to 102.89 N/m for stiffness. Bland–Altman plots showed no systematic bias for frequency values of the evaluated muscles in terms of the inter-rater reliability analysis (Figure S3). For stiffness values, except biceps brachii muscle for the left and right sides and left-side gastrocnemius muscle, no systematic bias was detected for the inter-rater reliability analysis (Figure S4).

## 4. Discussion

This study was designed to investigate the intra- and inter-rater reliabilities of myotonometer measurements in children with SMA Types I and II. Based on the study results, excellent intra-rater repeatabilities were found for the myotonometer to quantify biceps brachii, triceps brachii, rectus femoris, and gastrocnemius muscle tone and stiffness. Except for stiffness of the gastrocnemius muscle, which presented good inter-rater reliability, all inter-rater reliability results of the muscles presented excellent performance.

Lidström et al. reported moderate to high intra-rater reliability of rectus femoris muscle tone in children with cerebral palsy (age range 7–15.2 years, *n* = 15) under contracted (ICCs = 0.67–0.95) and relaxed (ICCs = 0.80–0.87) muscle conditions. In healthy children (age range 6.4–15.1 years, *n* = 15), intra-rater reliability for the tone of this muscle was found to show an ICC = 0.81–0.96 when contracted and ICC = 0.89–0.94 at rest [20]. Seo et al. reported intra-rater reliability of muscle-tone measurements in children with developmental disability in the biceps brachii and brachioradialis as 0.68 and 0.75, respectively, and those in the rectus femoris and the tibialis anterior as 0.78 and 0.75, respectively [21]. In our results, intra-rater reliability was excellent for muscle tone in children with SMA Types I and II with ICCs ≥ 0.83 for bilateral biceps brachii, triceps brachi, rectus femoris, and gastrocnemius muscles at rest. While our study results were similar to the ICCs of healthy children in the above-mentioned study [20], they were higher than those in the cerebral palsy group and children with developmental disability. So, it was assumed that the difference between the studies may be related to differences in pathology. In addition, we compared our result with Mucklet et al.’s study, which reported SEM and MDC values in adults. Mucklet et al. reported MDC values in 20 healthy subjects (age 28.95 ± 2.77 years) in the range of 2.4 Hz to 4.6 Hz with a moderate to excellent intra-rater reliability (ICC = 0.51–0.90) for resting muscle tone [19]. In this study, the best agreement in terms of resting tone of the bilateral gastrocnemius muscle was found with small SEM and MDC values. Repeatability with 95%CIs in the range of 0.59 to 0.99 was found for the resting tone of the left rectus femoris, bilateral triceps brachii, bilateral biceps brachii, and finally the right rectus femoris muscle. MDC values for muscle tone ranged between 1.07 Hz and 1.98 Hz in all examined muscles. One possible reason why ICC scores were lower and MDC and SEM were higher than in our study results may be that they performed a between-day intra-rater reliability examination. Our findings showed that MyotonPRO is reproducible with high reproducibility in the assessment of resting muscle tone in children with SMA Types I and II.

A study including children with spastic cerebral palsy reported the intra-rater reliability of myotonometer for biceps brachii muscle stiffness as 0.82 to 0.99 and for medial gastrocnemius muscle stiffness as 0.88 to 0.99 (age range 5–12 years, *n* = 10) [12]. Muckelt et al. reported that moderate, good, and excellent (ICCs = 0.57–0.98) repeatability was found for different body regions in adults with MDC values ranging from 40.5 to 140.6 N/m for between-day intra-rater reliability [19]. In our study, intra-rater reliability was excellent (ICCs ≥ 0.89) with 95% CI in the range of 0.70–0.99 for stiffness measurements from eight different sites for four muscles. The highest MDC scores were recorded for triceps brachii (124.74 N/m to 118.85 N/m) and the lowest for rectus femoris (31.29 N/m to 33.87 N/m). The SEM scores also showed a linear change with MDC. There is limited research on the SEM and MDC. Lower SEM and MDC values indicate higher device reliability. In this study, the low SEM and MDC values suggest a strong level of confidence in the measurement outcomes [16]. Repeatability of using MyotonPRO for stiffness value ranked from high to low for rectus femoris, biceps brachii, gastrocnemius, and triceps brachii muscles. These findings can serve as reference points for myotonometers and assist clinicians and researchers in detecting small changes in muscle properties. In light of these findings, the current study reported excellent repeatability results within the session using a myotonometer to quantify the tone and stiffness of different upper and lower extremity muscles of children with SMA Types I and II.

Previously, the inter-rater reliability of using myotonometer at seven different reference points in healthy adults has been demonstrated from moderate to excellent reproducibility for muscle resting tone (ICCs = 0.52–0.95, MDC= 1.6–5.0 Hz) and good to excellent reproducibility for stiffness (ICCs = 0.73–0.97, MDC= 40.9–127.9 N/m) [19]. Furthermore, Lidström et al. reported ICCs for rectus femoris tone in children with cerebral palsy as 0.62 to 0.89 under contracted and 0.57 to 0.75 under resting conditions. In healthy children, these values were reported as ICC = 0.73–0.95 under contracted and 0.87–0.92 under resting conditions [20]. Aarestad et al. examined the inter-rater reliability of the myotonometer used for measurements of the stiffness of the biceps brachii and medial gastrocnemius in children with spastic-type cerebral palsy. They found ICC values ranging from 0.74 to 0.99 for biceps brachii and 0.84 to 0.99 for medial gastrocnemius and reported a 98% level of agreement in inter-rater reliability coefficients between raters under all conditions [12]. In our study results, inter-rater reliability was excellent for all muscle resting tones (ICCs ≥ 0.86) and stiffnesses (ICCs ≥ 0.81). Inter-rater reproducibility was satisfactory for children’s resting muscle tone assessment with a range of 0.66 to 0.98 with 95% CI. The MDC values for resting muscle tone ranged from 0.59 Hz to 1.74 Hz in all examined muscles. The inter-rater reliability of MyotonPRO was high for biceps brachii, triceps brachii and rectus femoris stiffness measurements with a 95% CI in the range of 0.73–0.98. However, the assessment of right (0.55–0.92, 95% CI) and left (0.29– 0.88, 95% CI) gastrocnemius muscle stiffness may be considered to be performed with different motor point determinations. In this study, the reference site for measurements in the gastrocnemius muscle was determined as the point at which the muscle bulges the most (according to SENIAM sensor location guideline), which may have caused this result. Our results showed that MyotonPRO showed high reproducibility and reliability in the assessment of resting muscle tone in children with SMA. Also, we propose that the protocol in this study displayed high reproducibility for the use of MyotonPRO in the clinic.

Although it has been shown that the main affected center in SMA contains alpha motor neurons that are located in the anterior horn of the spinal cord, examining muscle mechanical properties may have important implications for a better understanding of potential peripheral mechanisms. Electrical impedance myography [33] or electromyography [3] reflects the denervation of muscles after low motor neuron degeneration in SMA; however, children’s toleration for this measurement may not be quite high. Gawel et al. confirmed that motor unit loss estimation obtained by the multipoint incremental motor unit number estimation (MUNE) method is a useful and easy tool to administer and is well-tolerated by children with SMA. The MUNE method is recommended to establish the electrophysiological diagnosis of SMA instead of using routine needle electromyography (EMG) [34]. In studies with adults, myotonometer results were found to be highly correlated with surface EMG and moderately to highly correlated with the modified Ashworth scale [26,35]. Since EMG is a time-consuming method and analyzing data obtained from EMG measurements can be difficult to perform, the myotonometer may have advantages compared to EMG. Myotonometer is simple to use and well tolerated by children, and also obtaining and interpreting data are quite easy to perform [36].

With an aim to observe peripheral changes easily, MyotonPRO can be an easy and quick assessment approach compared to EMG for quantifying skeletal muscle mechanical properties. This tool may help clinicians and researchers examine peripheral changes in the pathogenesis of SMA within the framework of skeletal muscle mechanical properties, to reach objective data on changes in skeletal muscle properties, and to observe changes in motor evaluation criteria, such as CHOP-INTEND (gold standard) that cannot be displayed with the plateau effect created by new generation treatments. Objective assessment criteria for clinicians working with SMA patients are quite limited.

The limitations of this study are as follows: First, the lack of possibility to address long-term reproducibility is due to the lack of between-day reliability assessments. Second, important variables that may affect muscle tone, stiffness, and endurance, such as ambient temperature and body temperature, were not controlled. Third, although our team recommended that participants be comfortable throughout the experiment, muscle contraction could not be precisely controlled without the use of electromyography. In future studies, this study’s limitations should be considered by controlling essential variables that may affect muscle tone, stiffness, and resilience, such as room temperature and electromyography. The ultrasound-guided validity and between-day reliabilities can be investigated with MyotonPRO in the upper and lower extremity muscles in children with SMA Types I and II.

## 5. Conclusions

In conclusion, the results of this research show that MyotonPRO is a reliable device for clinicians to quantify the mechanical properties of upper and lower extremity muscle tone and stiffness in individuals with SMA Types I and II aged between 1 and 4 years old. Nevertheless, these study results are limited only to session repeatability, and day-to-day reliability still needs to be examined. The high reliability in the repeatability and reproducibility of MyotonPRO in this study will contribute to future studies.

## Figures and Tables

**Table 1:** Demographic and clinical characteristics.

Characteristic	
Male sex, *n* (%)	17 (81%)
Age at evaluation in months, mean (SD)	32.38 (11.53)
Start date of the first symptoms, mean (SD)	2.80 (3.57)
SMA Type	
I	16 (76.2%)
II	5 (23.8%)
Medication	
Spinraza	3 (14.3%)
Spinraza and Zolgensma	15 (71.4%)
Risdiplam and Zolgensma	2 (9.5%)
Risdiplam, Spinraza and Zolgensma	1 (4.8%)
Nutrition type	
Nasogastric	3 (14.3%)
PEG	7 (33.3%)
Oral	11 (52.4%)
Presence of tracheostomy, *n* (%)	4 (19.0%)
Cough assist, *n* (%)	
Yes	16 (76.2%)
No	5 (23.8%)
CHOP-INTEND score	34.70 (18.47)
Need for BiBAP, *n* (%)	
Only during sleep	12 (57.1)
More than 16 h in a day	2 (9.5)
No	7 (33.3)

Abbreviations: SD, standard deviation; SMA, spinal muscular atrophy; CHOP-INTEND, Children’s Hospital of Philadelphia Infant Test of Neuromuscular Disorders; BiBAP, bilevel positive airway pressure; PEG, percutaneous endoscopic gastrostomy.

**Table 2:** MyotonPRO frequency results for intra-rater and inter-rater reliability.

Measurement Point	ICC (95% CI) Inter-Rater Reliability	ICC (95% CI) Intra-Rater Reliability	MDC Intra-Rater Reliability	MDC Inter-Rater Reliability	SEM Intra-Rater Reliability	SEM Inter-Rater Reliability
Left Biceps Brachii	0.92 (0.82–0.97)	0.93 (0.84–0.97)	1.64	1.54	0.59	0.55
Right Biceps Brachii	0.88 (0.70–0.95)	0.93 (0.85–0.97)	1.93	1.34	0.69	0.48
Left Triceps Brachii	0.95 (0.87–0.98)	0.93 (0.82–0.97)	1.64	1.74	0.59	0.62
Right Triceps Brachii	0.94 (0.85–0.97)	0.93 (0.85–0.97)	1.73	1.74	0.62	0.62
Left Rectus Femoris	0.90 (0.77–0.96)	0.95 (0.88–0.98)	1.19	0.83	0.43	0.30
Right Rectus Femoris	0.93 (0.83–0.97)	0.83 (0.59–0.93)	1.07	1.47	0.38	0.53
Left Gastrocnemius	0.87 (0.68–0.94)	0.96 (0.92–0.98)	1.98	0.92	0.71	0.33
Right Gastrocnemius	0.86 (0.66–0.94)	0.98 (0.96–0.99)	1.74	0.59	0.62	0.21

Abbreviations: ICC, interclass correlation coefficient; CI, confidence intervals; MDC, minimal detectable change; SEM, standard error of measurement.

**Table 3:** MyotonPRO stiffness results for intra-rater and inter-rater reliability.

Measurement Point	ICC (95% CI) Inter-Rater Reliability	ICC (95% CI) Intra-Rater Reliability	MDC Intra-Rater Reliability	MDC Inter-Rater Reliability	SEM Intra-Rater Reliability	SEM Inter-Rater Reliability
Left Biceps Brachii	0.90 (0.77–0.96)	0.94 (0.85–0.97)	49.78	40.69	17.96	14.68
Right Biceps Brachii	0.89 (0.73–0.95)	0.89 (0.73–0.95)	52.32	45.68	18.89	16.48
Left Triceps Brachii	0.92 (0.81–0.97)	0.93 (0.85–0.97)	118.85	102.09	42.87	36.83
Right Triceps Brachii	0.90 (0.75–0.96)	0.93 (0.84–0.97)	124.74	97.54	45.00	35.19
Left Rectus Femoris	0.94 (0.87–0.97)	0.97 (0.93–0.98)	31.29	23.51	11.28	8.48
Right Rectus Femoris	0.96 (0.91–0.98)	0.99 (0.97–0.99)	33.87	16.08	12.22	5.80
Left Gastrocnemius	0.71 (0.29–0.88)	0.96 (0.92–0.98)	116.07	30.71	41.87	11.08
Right Gastrocnemius	0.81 (0.55–0.92)	0.95 (0.89–0.98)	66.79	29.21	24.09	10.54

Abbreviations: ICC, interclass correlation Coefficient; CI, confidence intervals; MDC, minimal detectable change; SEM, standard error of measurement.

**Table 4:** Mean differences, upper and lower limits of agreement values of the evaluated muscles for frequency and stiffness parameters.

Measurement Point	Mean Difference (SD) (95% CI) Intra-Rater Reliability	Mean Difference (SD) (95% CI) Inter-Rater Reliability	LOA Intra-Rater Reliability		LOA Inter-Rater Reliability	
			Upper	Lower	Upper	Lower
Frequency (Hz)						
Left Biceps Brachii	0.05 ± 1.12 (−0.56–0.45)	0.06 ± 1.01 (−0.29–0.52)	2.14	−2.24	2.03	−1.91
Right Biceps Brachii	0.60 ± 1.27 (0.02–1.17)	0.65 ± 0.90 (0.24–1.07)	3.08	−1.88	2.42	−1.10
Left Triceps Brachii	0.70 ± 1.06 (0.21–1.19)	−0.19 ± 1.14 (−0.71–0.32)	2.78	−1.37	2.04	−2.42
Right Triceps Brachii	0.03 ± 1.14 (−0.86–0.17)	−0.17 ± 1.17 (−0.70–0.36)	1.89	−2.57	2.11	−2.46
Left Rectus Femoris	0.16 ± 0.82 (−0.21–0.53)	−0.20 ± 0.61 (−0.48–0.69)	1.76	−1.44	0.98	−1.40
Right Rectus Femoris	0.23 ± 0.67 (−0.07–0.54)	0.37 ± 1.17 (−0.15–0.91)	1.54	−1.07	2.67	−1.91
Left Gastrocnemius	0.31 ± 1.18 (−0.22–0.85)	0.08 ± 0.62 (−0.20–0.36)	2.63	−1.99	1.29	−1.13
Right Gastrocnemius	0.24 ± 1.11 (−0.26–0.75)	0.18 ± 0.38 (0.14–0.36)	2.41	−1.93	0.93	−0.55
Stiffness (N/m)						
Left Biceps Brachii	5.76 ± 35.63 (−21.98–10.45)	−0.21 ± 28.32 (−13.10–12.67)	62.11	−73.63	55.29	−55.72
Right Biceps Brachii	12.38 ± 32.57 (−0.24–27.20)	19.61 ± 31.06 (5.48–33.75)	76.21	−51.45	80.49	−41.25
Left Triceps Brachii	21.95 ± 75.80 (−12.55–56.46)	11.04 ± 62.97 (−17.61–39.71)	170.52	−126.61	134.46	−112.37
Right Triceps Brachii	27.71 ± 83.94 (−65.92–10.49)	35.66 ± 59.06 (8.77–62.55)	138.80	−192.23	151.42	−80.09
Left Rectus Femoris	2.61 ± 22.14 (−7.46–12.70)	−2.80 ± 15.71 (−9.96–4.34)	46.01	−40.77	27.98	−33.60
Right Rectus Femoris	2.52 ± 21.45 (−12.28–7.24)	1.66 ± 11.58 (−3.60–6.94)	39.51	−44.56	24.36	−21.03
Left Gastrocnemius	17.14 ± 59.16 (−9.78–44.07)	3.73 ± 20.52 (−5.60–13.08)	133.09	−98.81	43.95	−36.48
Right Gastrocnemius	2.90 ± 40.26 (−15.42–21.23)	10.52 ± 18.02 (2.31–18.72)	81.81	−76.00	45.84	−24.79

Abbreviations: Hz, Hertz; N/m, Newton per meter; CI, confidence intervals; SD, standard deviation; LOA, limits of agreement.

## Data Availability

Dataset available on request from the authors.

## Supplementary Materials

The following supporting information can be downloaded at: https://www.mdpi.com/article/10.3390/diagnostics14202300/s1, Figure S1: Bland–Altman plot analysis of frequency for inter-rater reliability analysis; Figure S2: Bland–Altman plot analysis of stiffness for inter-rater reliability analysis; Figure S3: Bland–Altman plot analysis of frequency for intra-rater reliability analysis; Figure S4: Bland–Altman plot analysis of stiffness for intra-rater reliability analysis.
