# Supplementary material for: Intersession Intra-Rater and Inter-Rater Reliability of Myotonometer for Upper and Lower Extremity Muscles in Children with Spinal Muscular Atrophy
Source: Diagnostics (Basel). 2024 Oct 16;14(20):2300. doi: 10.3390/diagnostics14202300 (PMC11506227; doi:10.3390/diagnostics14202300)
Supplement: Supplementary file 1 [file diagnostics-14-02300-s001.zip › diagnostics-3180125-supplementary.pdf]

Figure S1. Bland-Altman plot analysis of frequency for inter-rater reliability analysis.

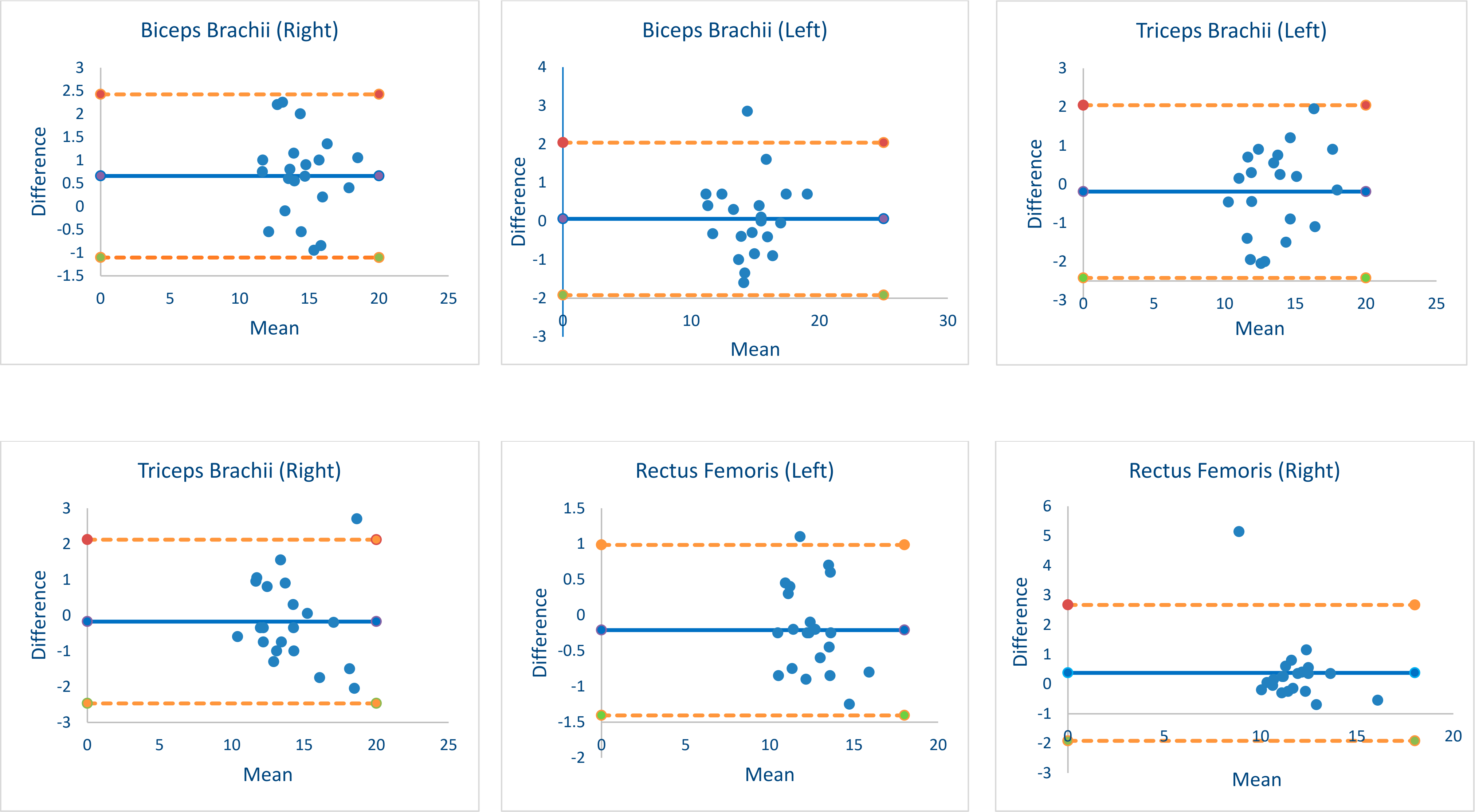

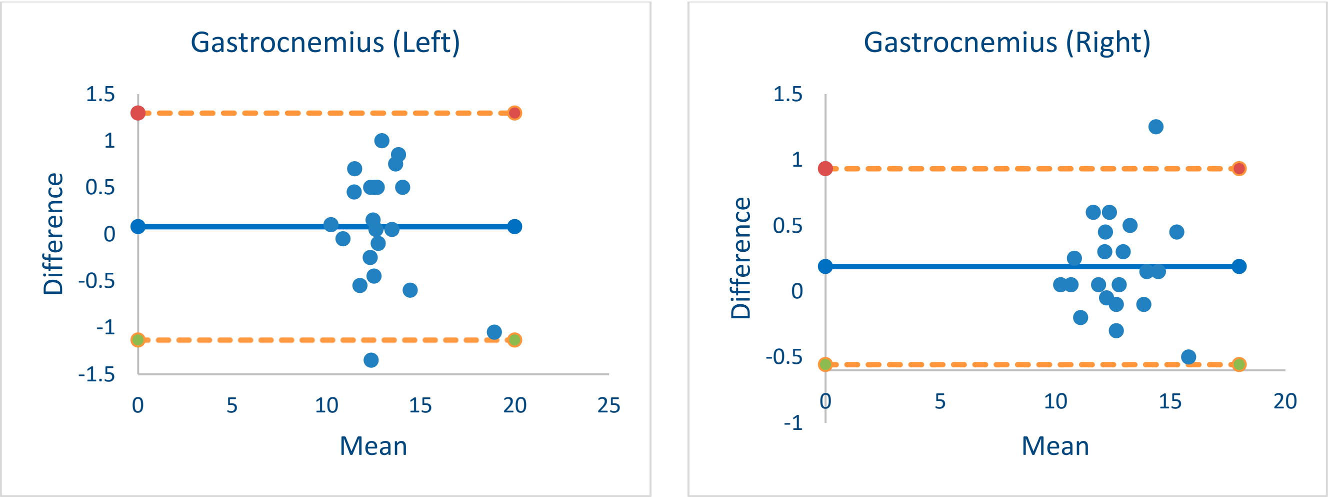
Gastrocnemius (Left)
Difference
Mean
1.5
1
0.5
0
-0.5
-1
-1.5
0
5
10
15
20
25
Gastrocnemius (Right)
Difference
Mean
1.5
1
0.5
0
-0.5
-1
0
5
10
15
20

Figure S2. Bland-Altman plot analysis of stiffness for inter-rater reliability analysis.

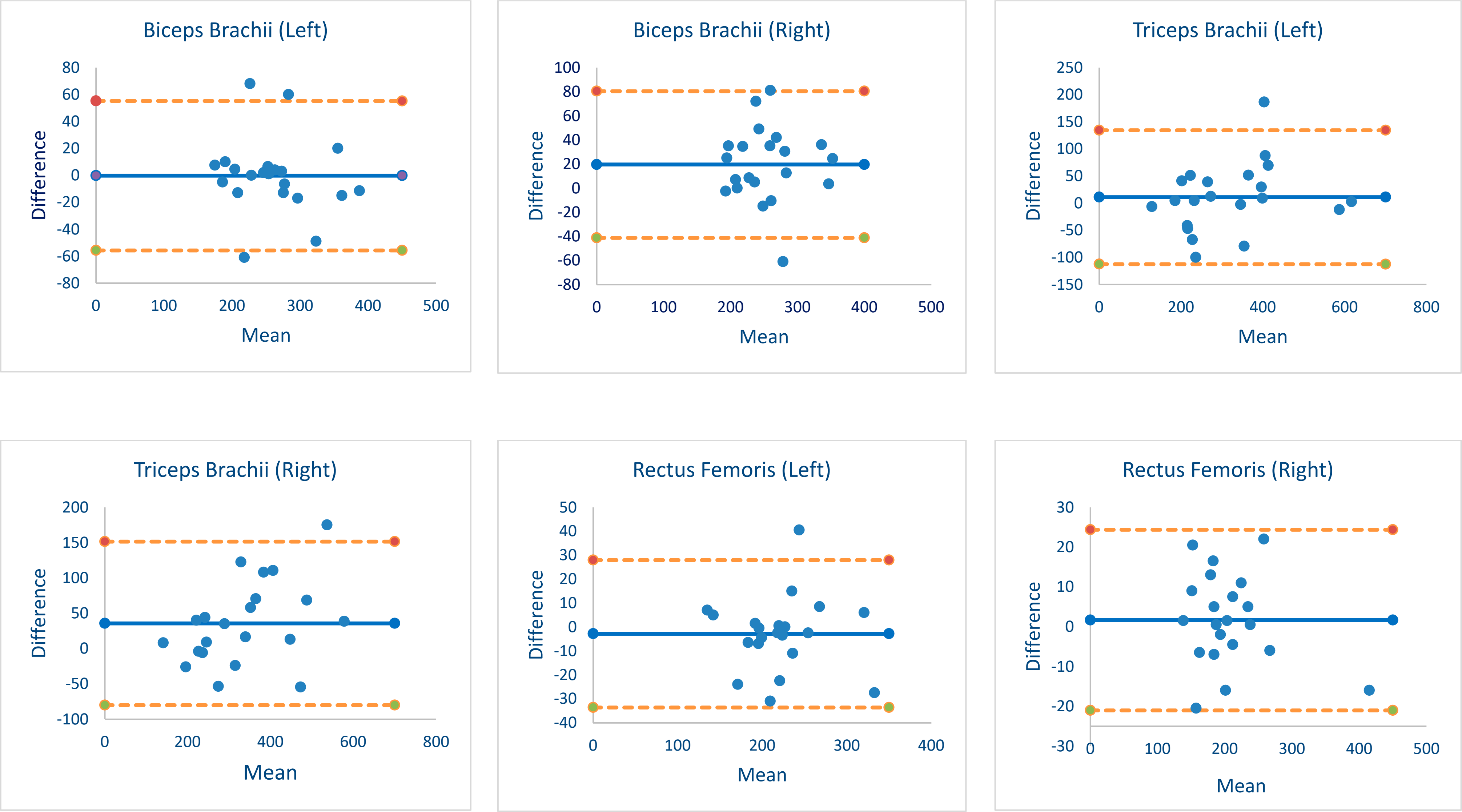

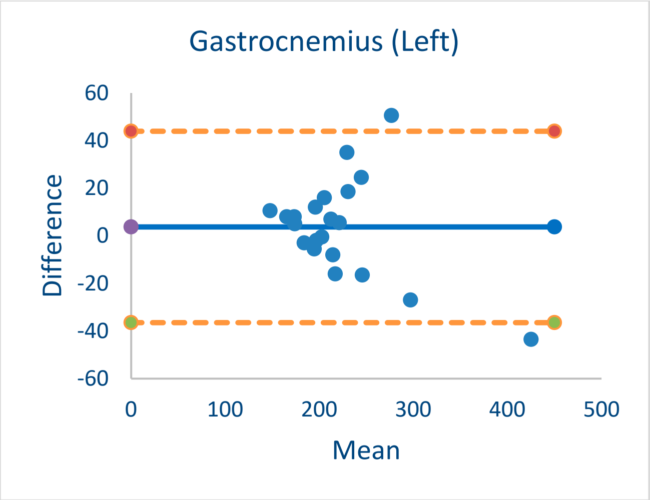
Gastrocnemius (Left)
60
40
20
0
-20
-40
-60
Difference
0
100
200
300
400
500
Mean

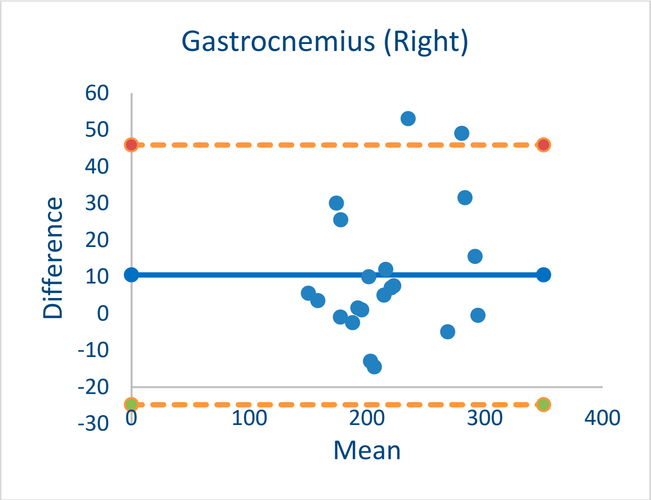
Gastrocnemius (Right)
60
50
40
30
20
10
0
-10
-20
-30
Difference
0
100
200
300
400
Mean

Figure S3. Bland-Altman plot analysis of frequency for intra-rater reliability analysis.

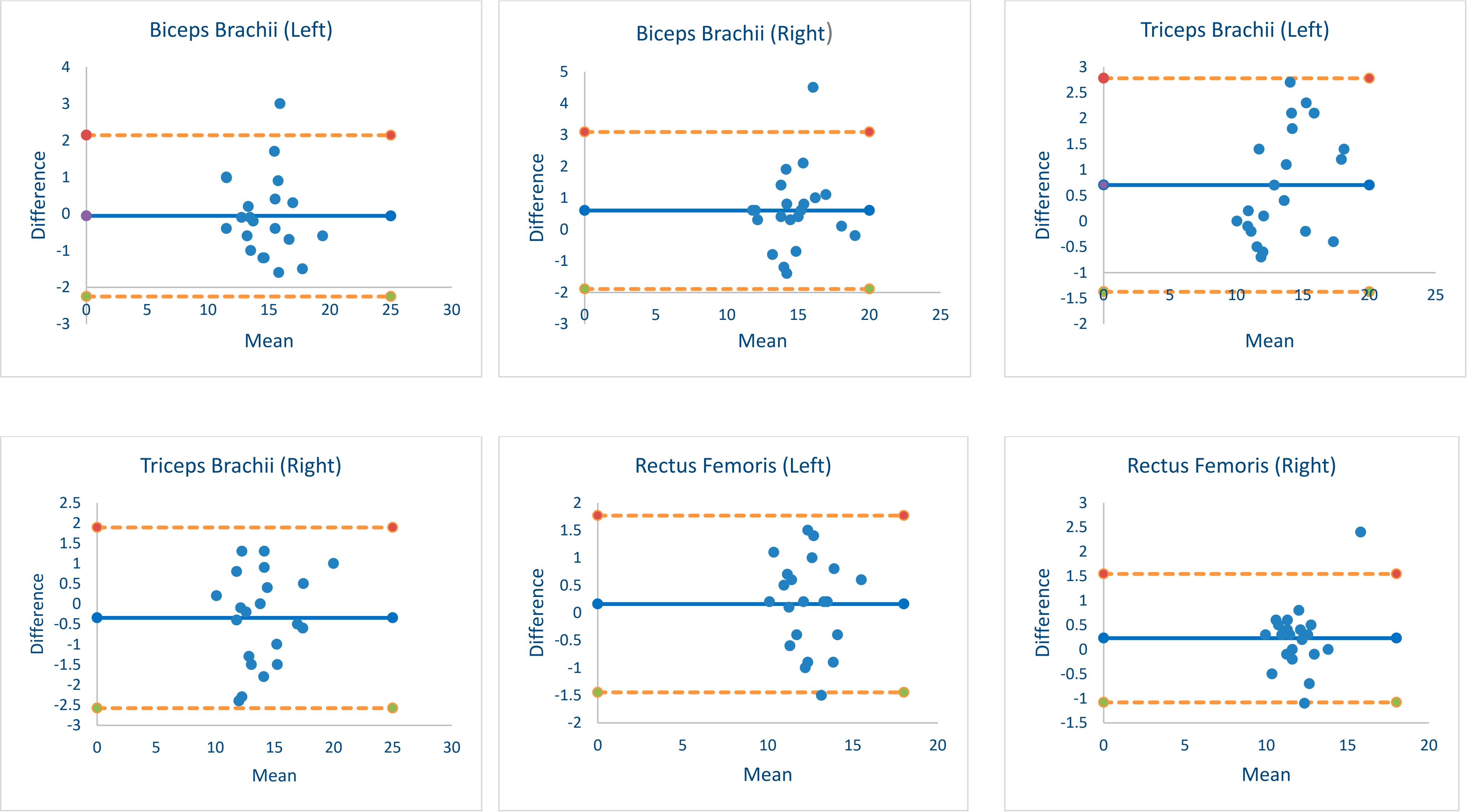

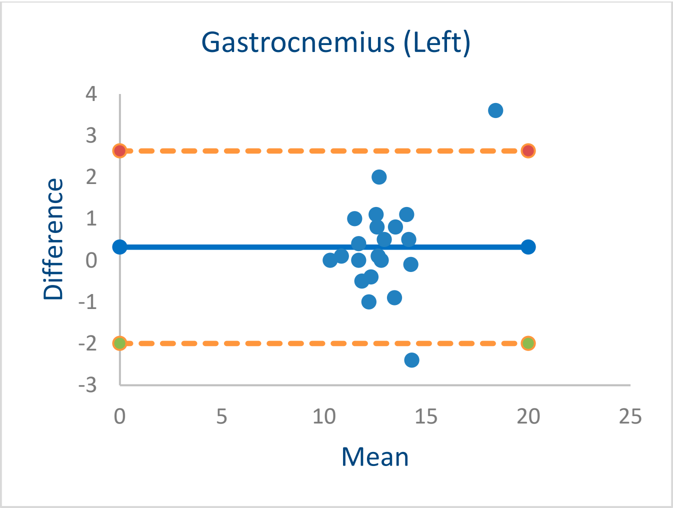
Gastrocnemius (Left)
Difference
Mean
4
3
2
1
0
-1
-2
-3
0
5
10
15
20
25

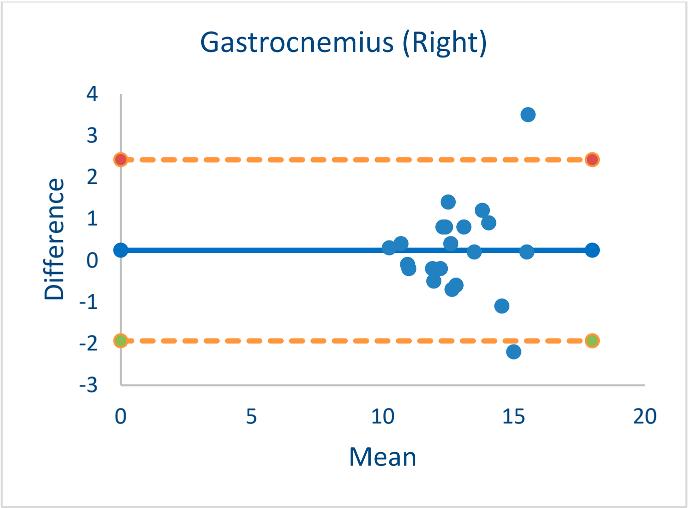
Gastrocnemius (Right)
Difference
Mean
4
3
2
1
0
-1
-2
-3
0
5
10
15
20

Figure S4. Bland-Altman plot analysis of stiffness for intra-rater reliability analysis.

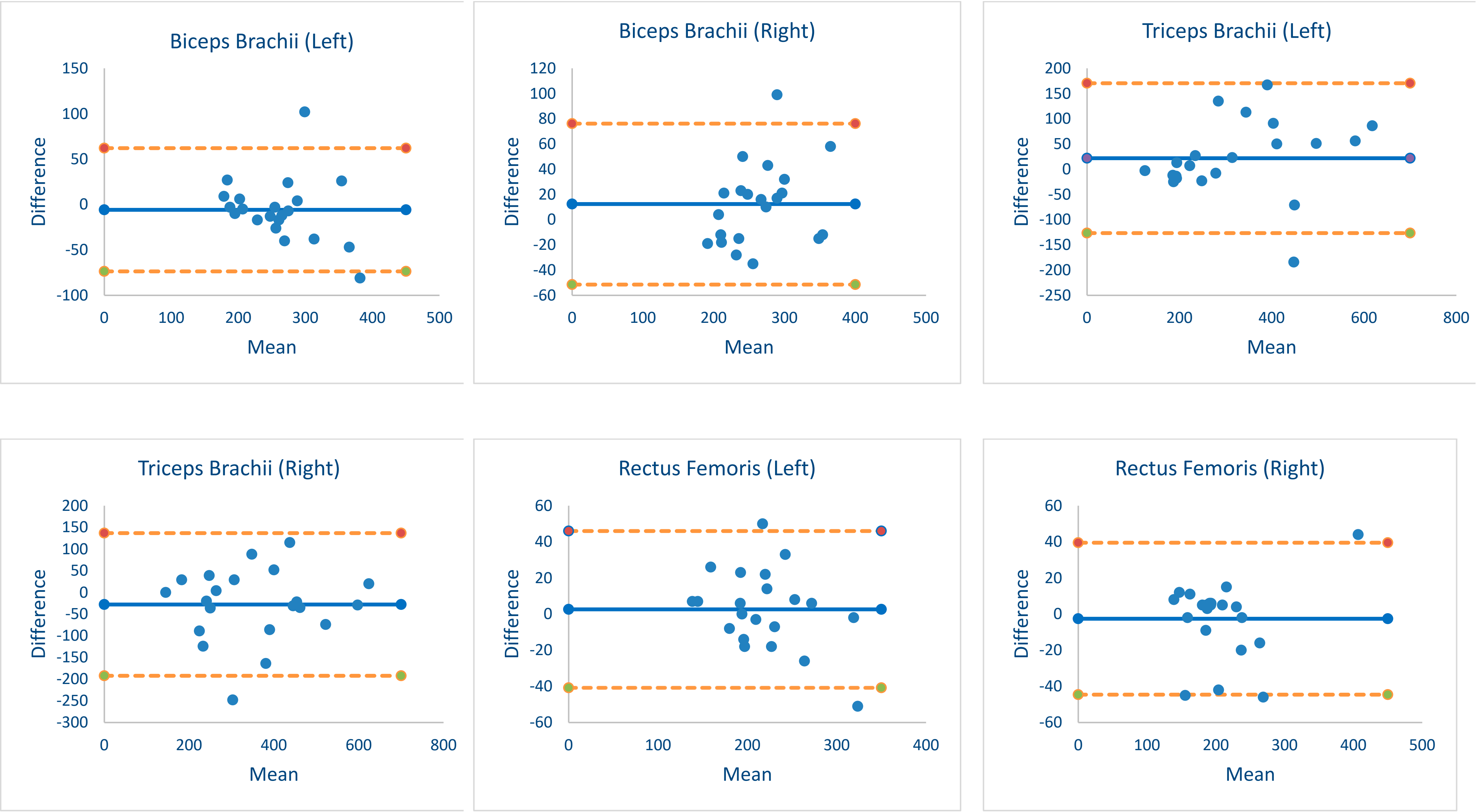

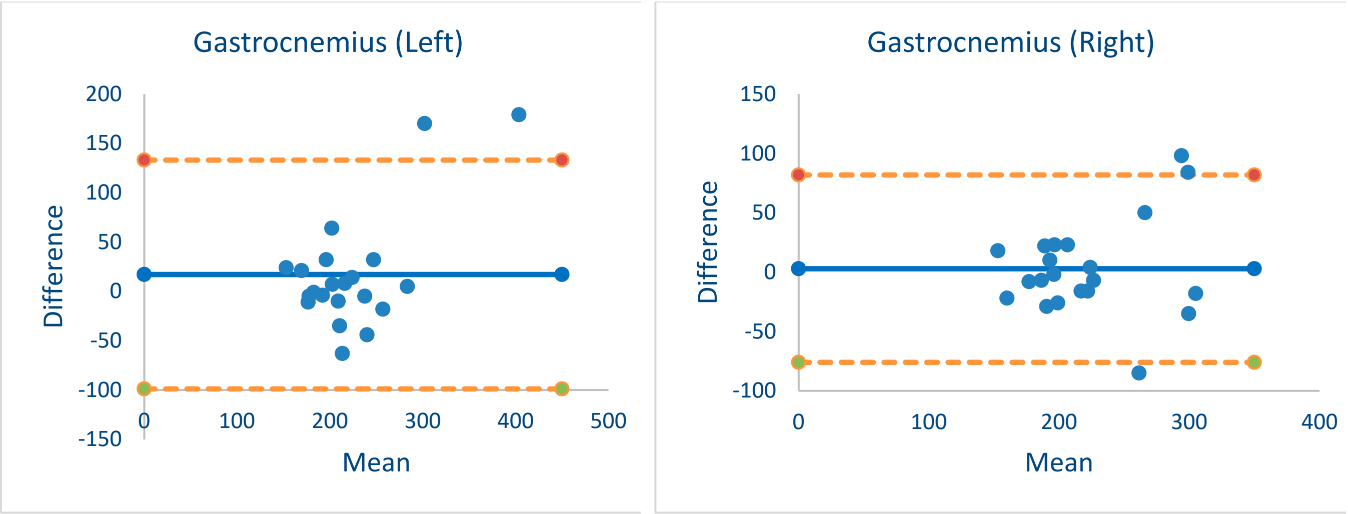

Gastrocnemius (Left)
200
150
100
50
0
-50
-100
-150
Difference
0
100
200
300
400
500
Mean
Gastrocnemius (Right)
150
100
50
0
-50
-100
Difference
0
100
200
300
400
Mean
